# Non-sedation versus sedation with a daily wake-up trial in critically ill patients recieving mechanical ventilation - effects on long-term cognitive function: Study protocol for a randomized controlled trial, a substudy of the NONSEDA trial

**DOI:** 10.1186/s13063-016-1390-5

**Published:** 2016-06-01

**Authors:** Helene Korvenius Nedergaard, Hanne Irene Jensen, Mette Stylsvig, Jørgen T. Lauridsen, Palle Toft

**Affiliations:** Department of Anesthesiology and Intensive Care, Lillebaelt Hospital, Skovvangen 2-8, DK-6000 Kolding, Denmark; Haugstedgaardsvej 5, 5230 Odense M, Region of Southern Denmark; Centre of Health Economics Research, Department of Business and Economics, University of Southern Denmark, Campusvej 55, 5230 Odense M, Denmark; Department of Anesthesiology and Intensive Care, Odense University Hospital, Sdr. Boulevard 29, 5000 Odense C, Denmark

**Keywords:** Critical illness/rehabilitation, Intensive care, Critical care/methods, Cognitive disorders, Delirium

## Abstract

**Background:**

The effects of non-sedation on cognitive function in critically ill patients on mechanical ventilation are not yet certain. This trial is a substudy of the NONSEDA trial where critically ill patients are randomized to non-sedation or to sedation with a daily wake-up attempt during mechanical ventilation in the intensive care unit (ICU).

The aim of this substudy is to assess the effects of non-sedation versus sedation with a daily wake-up attempt on long-term cognitive function.

**Methods:**

This is an investigator-initiated, randomized, clinical, parallel-group, superiority trial, including 200 patients.

Inclusion criteria will be adult patients who are intubated and on mechanical ventilation with an expected duration of more than 24 hours. Exclusion criteria will be patients who are comatose at admission and patients with conditions requiring therapeutic coma (i.e., severe head trauma, status epilepticus, patients treated with therapeutic hypothermia and patients with severe hypoxia).

The experimental intervention will be non-sedation supplemented with pain management during mechanical ventilation. The control intervention will be sedation with a daily wake-up attempt.

The primary outcome will be cognitive function 3 months after discharge from intensive care.

The secondary outcomes will be the results of seven specific cognitive tests, performed 3 months after discharge from intensive care, and the association between hypoactive and agitated delirium during ICU admission and long-term cognitive function.

**Discussion:**

If non-sedation can improve long-term cognitive function, it could be an approach worth considering for a larger group of critically ill patients.

**Trial registration:**

The study has been approved by the relevant scientific ethics committee and is registered at ClinicalTrials.gov (ID: NCT02035436, registered on 10 January 2014).

**Electronic supplementary material:**

The online version of this article (doi:10.1186/s13063-016-1390-5) contains supplementary material, which is available to authorized users.

## Background

This current trial is a substudy of the multinational NONSEDA trial (ClinicalTrials identifier: NCT01967680) [1]. The aim of the NONSEDA trial is to assess the benefits and harms of non-sedation versus sedation with a daily wake-up attempt in critically ill patients in the intensive care unit (ICU). Seven hundred patients will be randomized to non-sedation versus sedation with a daily wake-up attempt.

This substudy concerns long-term cognitive function and will be based on 200 of the 700 NONSEDA trial patients. The 200 patients will be included and treated in the ICU at trial site Kolding, Denmark. They will be followed up by a neuropsychologist 3 months after discharge from the ICU to assess their cognitive function.

### Patient population

Approximately 30,000 patients (2–3 % of all hospital patients) are admitted to ICUs in Denmark every year. In 2013–2014 the 30-day mortality for ICU patients was 27.1 % [2]. An intensive care admission can have substantial consequences for patients and studies show that ICU survivors have a reduced quality of life and an increased mortality for several years after discharge [3].

### Current care and treatment

Patients on mechanical ventilation are continuously sedated as a part of the standard approach. The first ventilators were rather primitive and highly uncomfortable for the patients, making sedation necessary. As ventilators have become more and more sophisticated, and now allow a high degree of patient-ventilator interaction and relative comfort, lighter levels of sedation are possible. Numerous trials have documented the beneficial effects of less sedation, namely shorter duration of mechanical ventilation, lower morbidity and shorter length of stay in the ICU and in hospital [4–10].

Critical illness affects both body and mind. Ehlenbach et al. analyzed 14-year follow-up data from a large cohort of older adults, and found that those who experienced acute care hospitalization and critical illness had a greater likelihood of cognitive decline compared with those who had no hospitalization [11]. Several studies document long-term cognitive impairments after, for example, acute respiratory distress syndrome or sepsis [12, 13]. The multicenter BRAIN-ICU study investigated long-term cognitive function in 821 patients after critical illness and found that 40 % of the patients had significant cognitive impairment at 3 months post ICU stay and 34 % at 12 months post ICU stay [14].

Delirium is a known risk factor for long-term cognitive impairment, and a common complication of critical illness [15–18]. Delirious patients can be either agitated, hypoactive or a mixture of the two. The type of delirium might affect cognitive function differently. For example, van den Boogaard found that those with hypoactive delirium had a higher mortality compared to agitated delirium, but might have a better long-term cognitive function [16]. It is difficult, if not impossible to diagnose delirium in sedated patients and it is, therefore, not surprising that our group, in a previous trial, observed a higher incidence of agitated delirium in non-sedated patients compared to sedated patients [5]. The patients in this previous trial were not assessed for long-term cognitive function.

The effect of non-sedation on long-term cognitive function has not been established, but several studies indicate that less sedation is not associated with impaired long-term cognitive function. Jackson et al. conducted a randomized trial, comparing daily spontaneous awakening trials to sedation per usual care [19]. They did not find adverse cognitive outcomes in the more awake patient group. Neither a recent meta-analysis nor the BRAIN-ICU study found that low sedation levels were associated with a higher incidence of cognitive dysfunction [20].

## Methods

### Aim and hypotheses

The aim of this randomized clinical trial is to assess the effects of non-sedation on cognitive function following ICU discharge.

Our primary hypothesis is that non-sedation compared with sedation and a daily wake-up trial will lead to a better long-term cognitive outcome.

We also hypothesize that both agitated and hypoactive delirium are negatively correlated with long-term cognitive function.

### Design

The trial is a substudy in the NONSEDA trial [1]. The NONSEDA trial is an investigator-initiated, randomized, clinical, parallel-group, multinational, superiority trial designed to include 700 patients from at least six ICUs in Denmark, Norway and Sweden. This substudy will be based on 200 of these 700 patients, namely those who are included at trial site Kolding, Denmark.

### Randomization

Patients will be randomized to one of the two groups within 24 hours after intubation. If the patient arrives intubated from another ICU, the patient will be randomized within the first 24 hours after arrival. The randomization will be carried out centrally by the Copenhagen Trial Unit according to a computer-generated allocation sequence with a variable block size, kept concealed from investigators at the clinical sites.

The allocation sequence will be stratified by center, age (up to 65 years or older) and the presence of Shock entails a systolic BP below 70 mmHg.

The 200 patients for this substudy will be all the patients included at trial site Kolding, Denmark. Since we will stratify for center, we will obtain an equal distribution of patients.

### Blinding

Due to the nature of the trial interventions, it will not be possible to blind the ICU staff or the participants to the individual participants’ randomization status. All other parties in the trial, including the neuropsychologist who conducts all the follow-up assessments, will be blinded. The statistical analyses will be conducted blinded with the two intervention groups coded as, e.g., A and B.

### Inclusion criteria

- Age 18 years or older
- Receiving endotracheal intubation
- Expected time on ventilator longer than 24 hours
- Informed consent obtained

### Exclusion criteria

- Severe head trauma where therapeutic coma is indicated
- Therapeutic hypothermia where therapeutic coma is indicated
- Status epilepticus where therapeutic coma is indicated
- Previous participation in this trial (during previous ICU admission)
- Transferral from another ICU with admission for more than 48 hours
- Comatose at admission
- Severe hypoxia (partial pressure of oxygen in arterial blood/fraction of oxygen in inspired air (PaO_2_/FiO_2_) ≤9) where sedation might be necessary for oxygenation or the need to position the patient in the prone position

### Trial site and personnel

The trial site is the Intensive Care Unit, Lillebaelt Hospital, Kolding, Denmark; a mixed medical and surgical ICU with 11 ICU beds and three intermediate care beds. The unit treated 998 patients in 2014 and 988 patients in 2015. The trial personnel will be doctors and nurses working in Kolding ICU. The personnel are already used to working with non-sedation and handling awake, mechanically ventilated patients as well as sedated patients with daily wake-up trials. The trial group will monitor the clinical work and, if needed, provide supplementary training in non-sedation and daily wake-up trials, both in theory and by supervised practice.

### Interventions

**Fig. 1:**
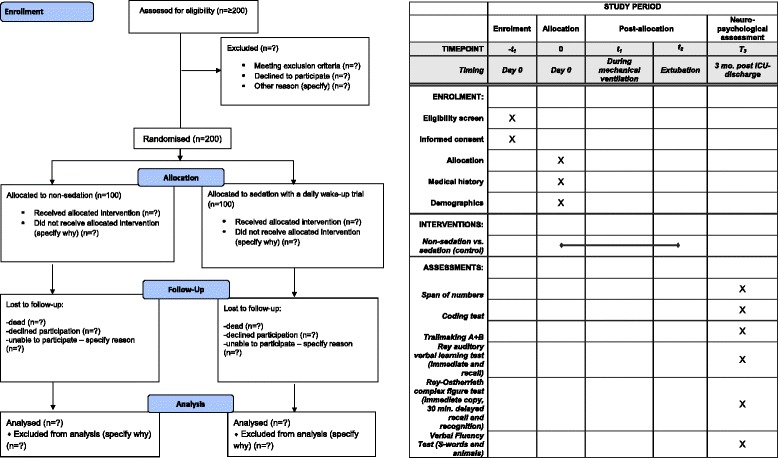
SPIRIT time schedule. Time schedule of enrollment, intervention, and follow-up assessment

As described in the NONSEDA trial protocol, the intervention consists of non-sedation supplemented with pain management versus sedation with a daily wake-up trial. In this substudy we will investigate how non-sedation versus sedation with a daily wake-up trial affects physical function after ICU discharge. For details about the interventions please see the NONSEDA trial protocol [1] (Fig. 1).

### Outcomes

The *primary outcomes* will be:

- Cognitive function 3 months after discharge from ICU, measured as a composite cognitive score. The score for each patient will be diagnosed by the neuropsychologist
- Number of patients with cognitive impairment in the two groups, as defined by Girard et al. [15]:
  - ◦ *mild to moderate cognitive impairment* if participants had either two cognitive test scores at 1.5 standard deviations (SD) below the mean or one cognitive test score at 2 SD below the mean
  - ◦ *severe cognitive impairment* if participants had three or more cognitive test scores at 1.5 SD below the mean or two or more cognitive test scores at 2 SD below the mean

The *secondary outcomes* will be:

Cognitive function, assessed by following neuropsychological tests:

- Span of numbers
- Coding test
- Trailmaking A + B
- Rey auditory verbal learning test (immediate, recall)
- Rey-Ostherrieth complex figure test (immediate)
- Rey-Ostherrieth complex figure test (delayed recall, recognition)
- Word-finding test (S-words and animals)

Effect of delirium during ICU admission:

- Hypoactive delirium, measured as the association between the occurrence of hypoactive delirium (Richmond Agitation-Sedation Scale (RASS) ≤0, Confusion Assessment Method for the Intensive Care Unit (CAM-ICU)-positive) and cognitive function after 3 months
- Agitated delirium, measured as the association between the occurrence of agitated delirium (RASS ≥ +2, CAM-ICU-positive) and cognitive function after 3 months

### Safety

There is no known risk associated with participation in the substudy. As a part of the NONSEDA trial protocol we register accidental extubation requiring re-intubation within an hour, and accidental removal of a central venous line requiring reinsertion within 4 hours, as serious adverse events and an interim analysis will be performed.

### Inclusion of patients

Patients can be admitted to the ICU either from other wards at the same hospital or transferred from an ICU in another hospital. If they are admitted from within the same hospital, they are either not intubated or have been intubated within a very short time: for example, during pre-hospital care. Patients will be included in the study within 24 hours from intubation.

A few patients will be transferred from an ICU in another hospital, and they will very often be intubated. They can be included in the trial if the stay in the other ICU was shorter than 48 hours. In the time leading up to inclusion and randomization, it will vary as to whether patients are sedated or not, depending on the particular clinician on duty and traditions at the particular hospital.

### Ethics, consent and permissions

The protocol has been approved by the Regional Scientific Ethical Committees for Southern Denmark (ID: S-20130025). We will obtain informed consent from the patients who are sufficiently awake; otherwise the informed consent will be obtained from the closest relative and the patient’s general practitioner, or alternatively the Medical Health Office. The trial is registered at ClinicalTrials.gov, ID: NCT02035436 (registration date 10 January 2014). Please see supplement material for SPIRIT-checklist (Additional file 1) and original consent forms (in Danish, Additional file 2).

When patients are contacted the first time concerning participation in the study, they will be in the ICU. Verbal and written information will be given by the trial coordinator (HKN) or the study nurse. Patients are informed about the rights to assistance and the possibility of reflection time. Patients will be considered competent if they are awake and not delirious (CAM-ICU-negative). The competent patients will give consent after a period of reflection time of up to several hours. If patients are not awake and not competent because of their illness, surrogate consent will be obtained from a close relative and the patient’s private practitioner, or alternatively the Medical Health Office. The consent of a relative relies on the patient’s presumed attitude to participation in clinical trials. The connection between the relative and the patient will appear in the surrogate consent form. Like the patient, the relative will also be given time of up to several hours to make the decision.

If, for any reason, a patient or their relative no longer wishes to participate in the trial, they will be asked for permission to use the previously obtained data in order to obtain data from electronic patient files for the rest of the trial period, and to invite the patient to the 3-month follow-up.

### Data management

An electronic Case Record Form (eCRF) for the NONSEDA trial in OpenClinica has been developed in cooperation between the coordinating investigator and a data manager at the Copenhagen Trial Unit. Daily access to the eCRF will be possible around the clock whereby data can be continuously entered for all the randomized patients.

The coordinating investigator will monitor the data input and will contact the primary investigator if data are missing on one or more randomized patients, in order to correct or complement inputs to optimize data quality.

### Data collection

All patient data during admission originate from medical records included in the Critical Information System (CIS) or other electronic patient files.

Before contacting any patient after discharge, we will check with national central person registrations to assure that the patient is not deceased. The process for establishing the follow-up will be as follows: approximately 14 days prior to the 3-month follow-up we will send a letter to the participant with an invitation to participate. If the patient does not respond, we will send a new letter repeating the invitation. If there is still no response, we will telephone the patient, repeating the invitation to participate in the follow-up and clarify any potential misunderstandings concerning transportation or the like. If the patient declines to come to the hospital, we will offer to come to the patient’s home and do the follow-up there.

The neuropsychological assessment will take place in a quiet room, away from busy wards and free from disturbances. Drinks and snacks will be provided as well as time for resting, if needed.

### Power estimation

Estimation of power for the primary outcome: 200 patients will be included, and the estimated 90-day mortality for this group of patients is 40 %, leaving an estimated 120 patients alive 3 months after discharge from the ICU. We assume a participation in the follow-up assessment at 75 %, thus leaving 90 patients to complete the interview, 45 in each group. With 45 patients in each group, and aiming at the difference in composite score means being statistically significant at a 5 % level, the difference in composite score means should be at least 0.41 times the standard deviation of the composite score. Then, using a simple normal test for comparison of two independent means, a 5 % significance in difference implies:

$$ \Delta =1.96*\sqrt{V}*\sqrt{45+45/45*45}=0.41*\sqrt{V}, $$

where ∆ denotes the difference in composite score, *V* denotes the variance for the composite score, which is assumed to be equal in the two groups, and *N* = 45, the sample size per group denotes the sample size per group.

### Statistical analysis plan

All continuous normally distributed outcome data will be described by mean, mean difference, standard deviation (SD) and range. Following Rasmussen et al. standard normal tests will be used to analyze differences in composite scores between the two groups [21]. However, multiple regression will be added to handle repeated measurements and to account for differences in composition of the two groups due to the absence of matching. Analyses will be performed as intention-to-treat.

All patients are followed up for at least 3 months after discharge via the electronic eCRF, Social Security Register and the National Patient Register. Missing data will be handled in accordance with multiple imputation procedures if missing data are greater than 5 % and Little’s test is statistically significant [22]. The imputation result will be considered the primary overall result but per-protocol analyses will also be presented. Extreme outliers will be identified and, if necessary, excluded.

All raw *p* values and confidence intervals of all outcome comparisons between the two groups will be presented. A *p* value <0.05 will be considered statistically significant in all analyses.

Statistical analysis of the data will be done using STATA 14 (Table 1).

**Table 1:** Cognitive tests used

Test	Description	Cognitive domain evaluated
Span of numbers [28]	Repeating a progressively longer sequence of numbers, first forwards, then backwards, then naming digits in numerical order	Attention/concentration
Coding test [28]	Translating numbers into figures using an answer key	Mental pace
Trailmaking A [29]	Drawing a line between consecutive numbers during a timed period	Mental pace
Trailmaking B [29]	Drawing a line between alternating numbers and letters during a timed period	Executive function
Rey auditory verbal learning test (immediate and recall) [30]	Repeating 15 words 5 times after hearing them aloud, and then again after 30 min as recalled	Verbal learning/memory
Rey-Ostherrieth complex figure test (immediate copy) [30]	Copying a drawing of a complicated geometrical figure while looking at it	Visual construction
Rey-Ostherrieth complex figure test (30-min delayed recall and recognition) [30]	Drawing the geometrical figure from memory after 3 and 30 min, and recognizing pieces from it from a catalog	Visual learning/memory
Verbal fluency test (S-words and animals) [31]	Naming as many words as possible beginning with S, and animals during 1 min each	Executive function/mental flexibility

## Discussion

The purpose of this randomized controlled trial, a substudy of the NONSEDA trial, is to investigate the effect of non-sedation during mechanical ventilation on cognitive function after discharge from the ICU. It is the first trial to our knowledge to investigate this, since the tradition and current standard management involves a higher degree of sedation.

We have designed this trial to be as realistic and generalizable as possible. The inclusion criteria are broad, the exclusion criteria are few and specific, the setting is an ordinary mixed ICU in a non-university hospital, patients are tended by multiple caregivers and the protocol for both the intervention and the control group is simple and relatively easy to follow in everyday care. This increases the generalizability and external validity of the trial.

We have chosen a very thorough testing of cognitive function. The same experienced neuropsychologist will test all patients to exclude interrater variability. Rather than completing questionnaires, patients will attend a 2-hour, semi-structured interview, based on validated and recognized cognitive tests. To include patients regardless of their pre-morbid status will cause some complications with regard to this follow-up assessment. There will be patients with dementia, stroke, terminal illness or conditions otherwise compromising them to an extent where they are simply not able to participate in the extensive interview. It is very important to limit the number of patients who not participating in the follow-up, since these might represent the outliers of the cognitive spectrum. We will make every effort to obtain a high participation rate. Rate and reason for not attending the follow-up will be thoroughly accounted for.

To blind as to whether patients are sedated or not is not possible. To minimize bias as much as possible, all major outcome assessments are performed by the same neuropsychologist who is blinded to the patients’ randomization status. It could be argued that the patients could spoil this blinding by revealing their randomization, but experience tells us that most patients are unaware as to which of the two groups they have been in. Memory can be severely affected by multiple factors encountered in the ICU: for example, critical illness in itself or sedatives. Furthermore, almost every patient will to some extent have experienced both sedation (at least during intubation) and non-sedation (at least just prior to extubation) which can also cause confusion.

Immobility and delirium are considered to be very high risk factors for cognitive impairment following critical illness. These are potentially modifiable conditions, and we feel certain that less sedation is a cornerstone for creating the optimal conditions for this [23]. In this trial we will investigate the importance of delirium on cognitive function. We assess mobilization and physical function in another separate substudy of the NONSEDA trial [24].

The goal of modern intensive care is not merely survival, but survival to a life worth living. In recent years, an awareness of so called Post-Intensive Care Syndrome (PICS) has arisen [25–27]. The syndrome comprises physical, psychological and cognitive sequelae after survival of critical illness. Despite awareness on the syndrome, little is known of about how to prevent it. Obviously the etiology for PICS must be multifactorial and highly variable since no two episodes of critical illness are identical. With the rising age of the population and treatment modalities becoming increasingly advanced, a greater number of fragile patients will survive critical illness. This highlights the need for knowledge on how to handle these patients as gently as possible in an attempt to preserve their premorbid level of health. With this trial it is our hope to clarify the “sedation pieces in the PICS puzzle.”

## Trial status

The trial is now actively recruiting patients. Inclusion of the first patient was on 9 January 2014 and inclusion of the last patient will be on 1 January 2017. As of 1 March 2016 we have included 148 of the 200 patients.

## Abbreviations

CAM-ICU, Confusion Assessment Method for the Intensive Care Unit; CIS, Critical Information System; eCRF, electronic Case Record Form; FiO_2_, fraction of oxygen in inspired air; ICU, intensive care unit; PaO_2_, partial pressure of oxygen in arterial blood, in mmHg; RASS, Richmond Agitation-Sedation Scale; SD standard deviation.

## Additional files

Additional file 1: SPIRIT 2013 Checklist: Recommended items to address in a clinical trial protocol and related documents. (PDF 130 kb) Additional file 2: Original consent forms, in danish. (PDF 259 kb)
