# Supplementary material for: Non-sedation versus sedation with a daily wake-up trial in critically ill patients recieving mechanical ventilation - effects on long-term cognitive function: Study protocol for a randomized controlled trial, a substudy of the NONSEDA trial
Source: Trials. 2016 Jun 1;17:269. doi: 10.1186/s13063-016-1390-5 (PMC4888731; doi:10.1186/s13063-016-1390-5)
Supplement: Additional file 2: — Original consent forms, in danish. (PDF 259 kb) [file 13063_2016_1390_MOESM2_ESM.pdf]

Patientinformation om deltagelse i et videnskabeligt projekt

Non-sedation sammenlignet med sedation og et dagligt opvågningsforsøg

Vi vil gerne spørge, om du vil deltage i et sundhedsvidenskabeligt forskningsprojekt.

Når man er indlagt på intensivafdeling, får man smertestillende medicin, så man er smertefri. Derudover er det på nogle intensivafdelinger rutine, at patienterne også bliver bedøvede og kun vækket af og til, så længe de har brug for respiratorbehandling. På andre intensivafdelinger er det rutine, at patienterne ikke bedøves, men naturligvis holdes smertefri, under respiratorbehandlingen.

Vi vil gerne undersøge, om det er bedst for patienter, der respiratorbehandles, at:

- være bedøvet, smertefri og blive vækket en gang dagligt - eller
- ikke være bedøvet, men smertefri.

Hvis du deltager i vores projekt, vil det være tilfældigt hvilken af disse behandlingsmetoder du behandles efter.

Der er ingen risiko ved at deltage i undersøgelsen. Hvis du skal være ikke-bedøvet, men har meget svært ved at tolerere at være vågen, vil du blive bedøvet ligesom den anden gruppe. Undersøgelsen stopper, hvis analyser viser, at den ene behandlingsmåde er den anden overlegen. Den øvrige behandling på intensivafdelingen foregår på sædvanlig vis, uanset hvilken gruppe man kommer i.

Hvis du deltager i projektet, vil du blive inviteret til en opfølgning, med en evaluering af din fysiske funktion og en neuropsykologisk interview-undersøgelse. Vi sender dig et brev med to spørgeskemaer og en invitation til opfølgningen tre måneder efter udskrivelsen. Hører vi ikke fra dig, ringer vi til dig efter et par uger.

Det er frivilligt, om du ønsker at deltage i undersøgelsen. Hvis du ikke ønsker at deltage, vil det ikke have indflydelse på din øvrige behandling. I forbindelse med informationen har du ret til at have en bisidder med, fx en af dine pårørende. Du har også mulighed for yderligere betænkningstid, hvis du ønsker det.

Du kan på et hvilket som helst tidspunkt vælge, at du ikke vil deltage i undersøgelsen mere. I så fald vil du blive spurgt, om vi må anvende de allerede opnåede data, om vi må søge informationer om dit forløb i elektroniske patientjournaler i resten af studieperioden og om vi må invitere dig til opfølgning efter 3 måneder.

Relevante myndigheder har adgang til videnskabelige data og patientjournaler i op til 10 år efter forsøget. Et halvt år efter forsøget kan du få oplysninger om resultatet ved henvendelse til Helene Korvenius Jørgensen eller Palle Toft. Palle Toft har taget initiativ til projektet, og undersøgelsen støttes økonomisk af Statens Strategiske Forskningsråd de næste 5 år. Undersøgelsen er godkendt af Den Videnskabsetiske Komite for Region Syddanmark.

Venlig hilsen

Helene Korvenius Nedergaard
Læge, ph.d-studerende
Anæstesiologisk-intensiv afd.
Kolding sygehus
6000 Kolding
Tlf.: 7636 3180
helene.korvenius.nedergaard@rsyd.dk

Palle Toft
Professor, overlæge, dr. med.
Anæstesiologisk-Intensiv Afdeling V
Odense Universitetshospital
5000 Odense C
Tlf.: 6541 3947
palle.toft@rsyd.dk

University of Southern Denmark

Region Syddanmark

Pårørende information

-til pårørende til patienter indlagt på intensiv afdeling
Non sedation sammenlignet med sedation og et dagligt opvågningsforsøg

Vi vil gerne spørge dig om lov til at din pårørende deltager i et sundhedsvidenskabeligt forskningsprojekt.

Når man er indlagt på intensiv afdeling, får man smertestillende medicin, så man er smertefri. Derudover er det på nogle intensivafdelinger rutine, at patienterne også bliver bedøvede og kun vækket af og til, så længe de har brug for respiratorbehandling. På andre intensivafdelinger er det rutine, at patienterne ikke bedøves, men naturligvis holdes smertefri, under respiratorbehandlingen.

Vi vil gerne undersøge, om det er bedst for patienter, der respiratorbehandles, at:

- være bedøvet, smertefri og blive vækket en gang dagligt - eller
- ikke være bedøvet, men smertefri.

Hvis din pårørende deltager i vores projekt, vil det være tilfældigt hvilken af disse behandlingsmetoder hun/hun behandles efter.

Der er ingen risiko ved at deltage i undersøgelsen. Hvis man skal være ikke-bedøvet, men har meget svært ved at tolerere at være vågen, vil man blive bedøvet ligesom den anden gruppe. Undersøgelsen stopper, hvis analyser viser, at den ene behandlingsmåde er den anden overlegen. Den øvrige behandling på intensivafdelingen foregår på sædvanlig vis, uanset hvilken gruppe man kommer i.

Hvis din pårørende deltager i projektet, vil han/hun blive inviteret til en opfølgning 3 måneder efter udskrivelsen, med en evaluering af den fysiske funktion og en neuropsykologisk interview-undersøgelse.

Det er frivilligt, om du ønsker at din pårørende deltager i undersøgelsen. Hvis du ikke ønsker at han/hun deltager, vil det ikke have indflydelse på den øvrige behandling. I forbindelse med informationen har du ret til at have en bisidder med. Du har også mulighed for yderligere betænkningstid, hvis du ønsker det.

Du kan på et hvilket som helst tidspunkt vælge, at din pårørende ikke skal deltage i undersøgelsen mere. I så fald vil du blive spurgt, om vi må anvende de allerede opnåede data, om vi må søge informationer om forløbet i elektroniske patientjournaler i resten af studieperioden og om vi må invitere din pårørende til opfølgning efter 3 måneder.

Relevante myndigheder har adgang til videnskabelige data og patientjournaler i op til 10 år efter forsøget. Et halvt år efter forsøget kan du få oplysninger om resultatet ved henvendelse til Hanne Tanghus Olsen eller Palle Toft. Palle Toft har taget initiativ til projektet, og undersøgelsen støttes økonomisk af Statens Strategiske Forskningsråd de næste 5 år. Undersøgelsen er godkendt af Den Videnskabsetiske Komite for Region Syddanmark.

Venlig hilsen

Helene Korvenius Nedergaard
Læge, ph.d-studerende
Anæstesiologisk-intensiv afd.
Kolding sygehus
6000 Kolding
Tlf.: 7636 3180
helene.korvenius.nedergaard@rsyd.dk

Palle Toft
Professor, overlæge, dr. med.
Anæstesiologisk-Intensiv Afdeling V
Odense Universitetshospital
5000 Odense C
Tlf.: 6541 3947
palle.toft@rsyd.dk

Informerende læge/sygeplejerske:

Navn: ______________________________

Adresse: ______________________________

Telefon: ______________________________

- □ Mundtlig information
- □ Folder: Før du beslutter dig.
- □ Folder om patienters rettigheder, ”Dine rettigheder som forsøgsperson i et biomedicinsk forskningsprojekt”

(S2)

Informeret samtykke til deltagelse i et sundhedsvidenskabeligt forskningsprojekt.

Forskningsprojektets titel: Non sedation sammenlignet med sedation og et dagligt opvågningsforsøg

Erklæring fra forsøgspersonen:

Jeg har fået skriftlig og mundtlig information og jeg ved nok om formål, metode, fordele og ulemper til at sige ja til at deltage.

Jeg ved, at det er frivilligt at deltage, og at jeg altid kan trække mit samtykke tilbage uden at miste mine nuværende eller fremtidige rettigheder til behandling.

Jeg giver samtykke til, at deltage i forskningsprojektet og har fået en kopi af dette samtykkeark samt en kopi af den skriftlige information om projektet til eget brug.

Forsøgspersonens navn: ____

Dato:________________ Underskrift:____

Hvis der kommer nye væsentlige helbredsoplysninger frem om dig i forskningsprojektet vil du blive informeret. Vil du **frabede** dig information om nye væsentlige helbredsoplysninger, som kommer frem i forskningsprojektet, bedes du markere her: ________(sæt x)

Ønsker du at blive informeret om forskningsprojektets resultat samt eventuelle konsekvenser for dig?:

Ja _____ (sæt x)   Nej _____ (sæt x)

Erklæring fra den, der afgiver information:

Jeg erklærer, at forsøgspersonen har modtaget mundtlig og skriftlig information om forsøget.

Efter min overbevisning er der givet tilstrækkelig information til, at der kan træffes beslutning om deltagelse i forsøget.

Navnet på den, der afgiver information:

Dato:________________ Underskrift:____

Projektidentifikation: (Fx komiteens Projekt-ID, EudraCT nr., versions nr./dato eller lign.)

S-20130025

(S7)

Stedfortrædende samtykke til deltagelse i et sundhedsvidenskabeligt forskningsprojekt.

Forskningsprojektets titel: Non sedation sammenlignet med sedation og dagligt opvågningsforsøg

Erklæring fra den person, som afgiver stedfortrædende samtykke:

Jeg har fået skriftlig og mundtlig information og jeg ved nok om formål, metode, fordele og ulemper til at give mit samtykke.

Jeg ved, at det er frivilligt at deltage, og at jeg altid kan trække mit samtykke tilbage uden at forsøgspersonen mister sine nuværende eller fremtidige rettigheder til behandling.

Jeg giver samtykke til, at ____(forsøgspersonens navn) deltager i forskningsprojektet og jeg har fået en kopi af dette samtykkeark samt en kopi af den skriftlige information om projektet til eget brug.

Oplysning om min tilknytning, som pårørende, til forsøgspersonen:

____

Navnet på den person, der giver stedfortrædende samtykke: ______________________________

Dato: ________________ Underskrift: ____

Ønskes information om forskningsprojektets resultat samt eventuelle konsekvenser for forsøgspersonen?:

Ja _____ (sæt x) Nej _____ (sæt x)

Erklæring fra den, der afgiver informationen:

Jeg erklærer, at der er afgivet mundtlig og skriftlig information om forsøget.

Navnet på det, der har afgivet information:

Dato: ________________ Underskrift: ____

Nedenstående udfyldes, når der ikke indgår personer under personligt værgemål:

Stedfortrædende samtykke fra forsøgspersonens praktiserende læge eller repræsentanten fra Sundhedsstyrelsens embedslægeinstitution:

Dato: ________________ Underskrift: ____

Projektidentifikation: (Fx komiteens Projekt-ID, EudraCT nr., versions nr./dato eller lign.)

S-20130025
